# Obstetrical Statistics

**Published:** 1850-12

**Authors:** 


### EDITORIAL DEPARTMENT.

*Obstetrical Statistics.*—The application of statistics to the study of the various subjects relating to health and disease, constitutes one of the most striking of the characteristics of modern medicine. In almost every department of the science the advantages flowing from numerical investigations are conspicuous. Of many of the points admitting of arithmetical demonstration, our knowledge, by this means, has been rendered full and exact, which heretofore was incomplete and dubious; certainty, in not a few instances, has taken the place of merely rational conjecture, and the false conclusions of speculation, or loose observations, have been displaced by results oftentimes not less unexpected than positive. Not only has truth been confirmed, and error disproved by statistical facts, but by the various methods of interpreting phenomena with the aid of figures, new relations have been disclosed, and new laws developed. Indeed, it has been discovered that the slate and pencil are important instruments of interrogating, as well as interpreting nature. In this way not only have fields already in possession been cultivated with greater success, but new territories have been explored and reclaimed, and the horizon which bounds the limits of our inquiries extended.

We have been led into this train of reflection by placing before us articles contained in two of our contemporaries, submitting the fruits of statistical researches in the province of obstetrics. The first of the articles referred to, may be found in the Am. Jour. of Med. Sciences, No. for Oct., 1850, and is entitled "Statistics of the Boston Lying-in-Hospital." By D. Humphreys Storer, M. D., one of the Physicians of the Massachusetts General Hospital. The other article is in the Philadelphia Med. Examiner, No. for October, 1850, and is entitled "Contributions to Obstetrics, with tabular views, and Miscellaneous Observations. By Henry A. Ramsay, M. D., Raysville, Georgia."

There are numerous points relating to obstetrics which admit of investigation by means of statistics, and respecting which, by series of observations sufficiently numerous and extended, our knowledge may be rendered more precise and comprehensive. Lying-in-hospitals, of course, offer a more favorable opportunity for collecting materials for enumeration, than private practice, yet the latter, with a little pains, may be made available for this purpose. The 'Contributions' by Dr. Ramsay, are the results of the analysis of the records of private cases. The habit of keeping notes of this class of cases, as well as of other classes, enhances our scientific interest in the obstetrical department of general practice, and, in this point of view, amply repays the trouble which it costs. We have, of late years, made it a duty to record the details of all cases of labor occurring under our observation, and have now collected between three and four hundred for analysis at some future period of leisure, when the number has become still greater. In this, as in other branches of statistical investigation, the results of different analyses by different persons, at different times, and in different places, are desirable. They will serve not alone as additions to numerical data, but to determine variations, which may be found to exist, incident to time, locality, and divers modes of practice.

With these remarks by way of introduction, we proceed to present a summary of the statistical results contained in the two articles alluded to, taking the liberty of condensing in so far as practicable, and omitting such portions as seem to us to possess comparatively little interest or importance. The cases analyzed by Dr. Storer, are all that have occurred at the Boston Lying-in Hospital, since the foundation of the Institution. The period is not stated in the Report. The reader, however, is led to infer that it embraces from ten to twenty years.

The whole number of deliveries is 451. The number of children in these deliveries is 456. Of 331 cases, 132 were males, and 199 females.

The youngest woman delivered was 16; the oldest 47.

The greatest number of times any of the women had been pregnant, was 16, which occurred in but one instance. The number of primipara was 193.

*No. of days from last menstruation.*—This is an important point with reference to the duration of pregnancy. We give the author's figures:—

| | | | | | | | | | | | | | |
|---|---|---|---|---|---|---|---|---|---|---|---|---|---|
| No. of days | 279 | 278 | 277 | 275 | 275 | 274 | 273 | 272 | 271 | 270 | 269 | 268 | 267 |
| No of cases | 7 | 8 | 11 | 9 | 4 | 6 | 6 | 6 | 8 | 20 | 2 | 4 | 2 |
| No. of days | 266 | 265 | 264 | 263 | 262 | 261 | 260 | 259 | 258 | 257 | 256 | 255 | 244 |
| No. of cases | 5 | 9 | 9 | 3 | 5 | 4 | 7 | 4 | 3 | 2 | 1 | 4 | 2 |
| No. of days | 253 | 252 | 250 | 249 | 246 | 245 | 244 | 243 | 242 | 240 | 236 | 228 | 220 |
| No. of cases | 4 | 2 | 6 | 1 | 2 | 2 | 2 | 3 | 1 | 11 | 1 | 1 | 1 |
| No. of days | 215 | 213 | 212 | 207 | 195 | 180 | 162 | 150 | 120 | 69 | 57 | | |
| No. of cases | 1 | 2 | 1 | 1 | 1 | 2 | 1 | 1 | 1 | 1 | 1 | | |

Total No. of cases 201.

The average number of days at which menstruation occurred previous to confinement, was 256, or 36 weeks.

In two cases menstruation occurred during each month of pregnancy. In the instance in which the patient was in her sixteenth pregnancy, menstruation had occurred regularly during all her pregnancies. She had had 12 children, and 3 miscarriages; had been married 19 years, and nursed all her children.

*The period of Quickening in* 158 *cases.*

| | | | | | | | | | | | | | | |
|---|---|---|---|---|---|---|---|---|---|---|---|---|---|---|
| Days before confinement | 60 | 83 | 86 | 90 | 93 | 96 | 100 | 106 | 109 | 110 | 111 | 113 | 114 | 116 |
| No. of women . . | 1 | 1 | 1 | 1 | 1 | 1 | 2 | 1 | 1 | 3 | 1 | 1 | 1 | 1 |
| Days before confinement | 118 | 119 | 120 | 121 | 122 | 124 | 125 | 126 | 128 | 129 | 130 | 131 | 132 | 133 |
| No. of women . . | 1 | 1 | 6 | 1 | 1 | 2 | 3 | 1 | 1 | 1 | 5 | 4 | 4 | 2 |
| Days before confinement | 134 | 135 | 136 | 137 | 138 | 139 | 140 | 142 | 143 | 144 | 145 | 146 | 147 | 148 |
| No. of women . . | 1 | 5 | 1 | 6 | 3 | 4 | 8 | 1 | 1 | 3 | 5 | 4 | 2 | 2 |
| Days before confinement | 149 | 150 | 152 | 153 | 154 | 156 | 157 | 158 | 159 | 160 | 161 | 163 | 164 | 165 |
| No. of women . . | 1 | 8 | 2 | 2 | 2 | 3 | 2 | 1 | 4 | 4 | 3 | 2 | 2 | 4 |
| Days before confinement | 166 | 168 | 169 | 171 | 173 | 174 | 175 | 176 | 180 | 186 | 187 | 188 | 192 | 199 |
| No. of women . . | 1 | 2 | 1 | 1 | 2 | 1 | 1 | 2 | 2 | 2 | 2 | 1 | 1 | 1 |

The least number of days in which quickening took place previous to confinement was 60—the greatest number of days previous to confinement was 199; of the former was one case, as well as also of the latter. The average number of days at which quickening occurred was 142.

A woman, aged 25, in her first pregnancy, stated "that she had never during her pregnancy experienced nausea or vomiting, nor felt quickening."

*The duration of Labor in* 433 *cases.*

| | | | | | | | | | | | | | | | | | | | | | | |
|---|---|---|---|---|---|---|---|---|---|---|---|---|---|---|---|---|---|---|---|---|---|---|
| Hours in labor | 1 | 2 | 3 | 4 | 5 | 6 | 7 | 8 | 9 | 10 | 11 | 12 | 13 | 14 | 15 | 16 | 17 | 18 | 19 | 20 | 21 | 22 |
| No. of women | 12 | 28 | 23 | 26 | 41 | 38 | 27 | 22 | 23 | 21 | 12 | 21 | 13 | 10 | 10 | 19 | 5 | 13 | 6 | 6 | 2 | 5 |
| Hours in labor | 24 | 25 | 26 | 27 | 28 | 30 | 31 | 32 | 33 | 34 | 35 | 36 | 37 | 40 | 41 | 42 | 45 | 48 | 53 | 58 | 70 | 74 | 88 |
| No. of women | 8 | 6 | 3 | 2 | 5 | 2 | 2 | 3 | 1 | 1 | 1 | 4 | 1 | 2 | 1 | 1 | 1 | 1 | 1 | 1 | 1 | 1 | 1 |

The least number of hours any woman was in labor was 1—the greatest number of hours was 88; of the former there were 12 cases, and of the latter 1. A greater number of labors occupied 5 hours than a longer time —and next in frequency were those completed in 6, 2, 7, 4, 3, 8, 10, and 12. The average number of hours was 11½.

In the 5 most protracted cases mentioned above, the mother did well in each instance, and in 3 of the cases the child was saved.

The case occupying 53 hours was one of mere inefficiency of pains.

That case which was terminated in 58 hours was rendered difficult by the presentation of "the right arm with the funis." The membranes broke without any pain being present, and the patient was not seen until several hours afterwards, when the arm and funis were found in the vagina. The child was immediately turned, but was *still.*

In the case in which labor was delayed 70 hours before its completion, a "sudden discharge of the liquor, without obvious cause," took place before the os uteri had become sufficiently dilated to expel the child.

In the case in which labor continued 74 hours, *the size of the child alone* appeared to be the only obstruction. The mother, twenty-five years old, had had two children, each of which, according to her account, weighed upwards of eleven pounds. She was in pretty good health, the presentation was natural, and she sustained her labor so well that I was unwilling to interfere—and eventually she gave birth, unaided, to a living male child, weighing twelve and a half pounds—the largest child which had been born in the institution.

In the remaining case, which occupied 88 hours, the left foot and head presented—the head considerably higher than the foot. "Some efforts were made to draw down the foot and push up the head, but finding the head most disposed to descend, the foot was supported during the pains, and the head came down."

*The time of Birth.*—In 428 cases, 214 occurred between the hours of 7 A. M. and 7 P. M.; and 214 cases between 7 P. M. and 7 A. M.

These results differ from those in 280 cases published by me in the *New England Quarterly Journal of Medicine and Surgery*, in 1843, and also 440 cases published by Dr. Metcalf, in the *American Journal of Medical Sciences*, for October, 1847—in each of which a larger number of births are stated to have occurred during the night than during the day.

*Weight of the Children in* 406 *cases.*

| | | | | | | | | | | | | | | | |
|---|---|---|---|---|---|---|---|---|---|---|---|---|---|---|---|
| Weight, | ¾lb. | 1 | 2 | 3 | 4 | 4¾ | 5 | 5¼ | 5½ | 5¾ | 6 | 6¼ | 6½ | 6¾ | 7 |
| Male, | 1 | | | 1 | | 2 | 2 | 2 | 9 | 3 | 8 | 1 | 18 | 8 | 17 |
| Female, | 1 | 1 | 1 | 1 | 2 | 1 | 3 | 2 | 6 | 5 | 9 | 10 | 9 | 7 | 14 |
| Weight, | 7¼lb. | 7½ | 7¾ | 8 | 8¼ | 8½ | 8¾ | 9 | 9¼ | 9½ | 9¾ | 10 | 10½ | 10¾ | 12½ |
| Male, | 11 | 27 | 15 | 28 | 7 | 11 | 2 | 14 | 2 | 12 | 5 | 3 | 1 | 1 | 1 |
| Female, | 10 | 25 | 14 | 24 | 6 | 11 | 7 | 7 | 2 | 3 | | 3 | | | |

The number of males in the above table was 2[illegible]2, whose aggregate total weight was 1669½ pounds; and the average weight of each was 7½ pounds.

The number of females was 184, whose aggregate total weight was 1309¾ pounds, and the average weight of each was 7$\frac{1}{9}$ pounds.

It will be observed that the weights of the above children fall considerably below those of the cases reported by me in the first volume of the *New England Journal of Medicine and Surgery*, and also of those registered by Dr. Metcalf in his paper previously referred to. I can account for this only by the fact that the great mass of his cases, if not every case reported in those communications, referred to American mothers, whereas the *vast majority* of the mothers of the children, included in the table here presented, were foreigners. That my reasoning is not fallacious, will be perceived by the following table:—

Of the 21 children which weighed 9 pounds, the mothers of 8 were Americans.

Of the 4 children which weighed 9¼ pounds, 3 had American mothers.

Of the 15 which weighed 9½ pounds, 9 were children of Americans.

Of the 5 weighing 9¾ pounds, 4 had American mothers.

Five of those which weighed 10 pounds, were children of Americans.

The one weighing 10¾ pounds, as well as that weighing 12½ pounds, were children of Americans.

In other words, of the 54 children which weighed 9 pounds and upwards, 31 had American mothers; although three-fourths of all the children born in the hospital had foreign parents.

The 4 children weighing less than 3 pounds, were premature and *still*. Those weighing 3 pounds were born alive; one of them "soon after leaving the hospital," which occurred ten days after its birth."

*Length of Funis.*—The most common length was two feet; the next in frequency was 26 inches; and the next 23 inches. The largest was 43 inches; and the shortest 4½ inches.

*Presentations in* 440 *cases.*

425 Vertex; 5 Breech; 2 Feet; 1 Face to pubis; 2 Hand to head; 1 Hand to face; 1 Foot and head; 1 Vertex and funis; 1 Arm and funis; 1 Placenta.

The cord was around the neck in 31 of 444 cases. It was *once* around the neck in 21 cases; *twice* in 7 cases; around the neck *twice* and passed under the right arm in one case; *thrice* around the neck in one case.

As regards the child in the 31 cases referred to, it did well in 28 cases. In several cases, it cried immediately upon being born.

In one case where it was resuscitated, the record states "funis twice around the neck, has no pulsation; child was washed in alcohol, and was well in a few minutes."

In the second case, the cord was very tightly coiled around the child's neck, so that previous to the exit of the shoulders, blood flowed freely from its nose and mouth; and the child was not perfectly resuscitated for nearly half of an hour.

Of the three still children, in which the funis encircled the neck, one was of a second labour, weighing 6¼ pounds, and nothing appears in the record to account for its death. "There was no pulsation in the cord, nor any other indication of life. The breast was sprinkled with alcohol, friction was employed, with artificial respiration, but without effect."

In the second case, the woman was thirty-seven hours in labour. Four days previous to her delivery, she was attacked *with bearing down pains followed by flooding*—and lost about a pint of blood at that time. The record does not show whether the motion of the fœtus was felt after that time or not.

In the third case, the umbilical cord was around the neck, and under the right axilla. This was a second pregnancy, and the woman thirty-four years of age. The child, a male, weighed 9½ pounds. Labour continued 28 hours, and was terminated by the exhibition of ergot.

The above cases serve to coroborate the opinion of Churchill, that when he cord is of the ordinary length, labour is not delayed by its being coiled around the neck of the child. And the experience of Cade* respecting the fœtus being destroyed by apoplexy, produced by the pressure of the funis around the neck—and also the experiments of Negrier "that the umbilical cord is both long enough and strong enough to produce strangulation in a new-born infant, by being twisted around its neck after the head is delivered."

NOTE.—As the question was asked by several gentlemen, upon the reading of this communication, "How did Negrier arrive at his conclusion?"—and as others upon perusing it may be disposed to make similar inquiries, I present the following details from the *Edinburgh Medical and Surgical*

---

* Reflexions et observations sur l'entabillement du cordon ombilical au'our du cou du fœtue.—*Encyclographie des Sciences Medicales.* Avril, 1841.

*Journal,* April 1st, 1841, page 556: "A girl of bad character was accused of having strangled her child by means of the unbilical cord, before it was completely expelled from the uterus. As there was a difference of opinion among the medical men as to the possibility of the umbilical cord possessing sufficient strength or length for this purpose, Dr. Negrier performed a number of Experiments for the purpose of ascertaining the strength of the cord, and measured it in 166 cases to arrive at its average length.

"Of the 166 cases, it was remarked that in 144 the umbilical cord floated free within the uterus; in 20 it was rolled around the neck of the child: in one it was round the shoulders; and in one between the thighs, the breast presenting in this case: 98 of the umbilical cords were not varicose, and 67 were varicose. As to length, 28 were 17 inches long; 112 were from 17 to 25½ inches long, and 26 above that length.

"The resistance of the umbilical cord was ascertained by attaching weights to one end of the cord until it ruptured, the weights being always attached to the placental extremity. About one-half of the cords were passed by their middle over a round bar, and weights attached till they gave way; the other half of the number were rolled once and a half round the same bar, covered with linen, so as to bring it to the diameter of a child's neck, when it was found that these supported a greater weight than the sound cords, and generally gave way at one of the various dilatations. The mean weight which these varicose cords supported before they gave way was eight pounds Troy; the most resistant supported fourteen pounds seven ounces. The medium resistance of the non-varicose umbilical cords was fourteen pounds four ounces Troy; but one cord required twenty-five pounds to rupture it.

"Dr. Negrier next made a few experiments to ascertain what weights suspended round the neck of an adult would produce such a degree of compression as to cause unpleasant feelings of strangulation. A weight of eight pounds was suspended to a cord passed once and a half round the neck, the back of the neck being upwards. The respiration was rendered difficult, and the brain strongly congested in two minutes. Vertigo commenced soon afterwards. The respiration, however, could be continued with difficulty. When the face was placed upwards, the effects of the congestion were more rapid; the respiration was much impeded, but was still possible; but Dr. Negrier thought that death would have resulted if this position had been maintained for a quarter of an hour.

"When the experiment was made with a weight of thirteen pounds, and the face downwards, rapid congestion of all the vessels of the head took place; the eyes became injected, and filled with tears; the respiration was very laborious, but was still possible. It was, however, dangerous to continue the experiment for two minutes.

"When the same experiment was repeated, but with the face looking upwards, the strangulation was almost complete. Respiration was so impeded that Dr. Negrier thinks death would have resulted in less than five minutes.

"From these facts, he infers that the umbilical cord is both long enough and strong enough to produce strangulation in a new-born infant, by being twisted round its neck after the head was delivered. A force applied to a cord equal to thirteen pounds, would strangle an adult in five minutes, and a much less force would strangle a child.

*Instrumental Deliveries.*—In 451 cases, eight were delivered by the forceps and two by craniotomy.

Of those delivered by the forceps, six were in cases of the first pregnancy, one in a second pregnancy, and one in a fifth pregnancy.

Five of the eight children were born alive; of the remaining three, one patient was aged 32; this was her second pregnancy, and the child presented with the face to the pubis. After a tedious labor of twenty-five hours, a male child was delivered weighing 6½ pounds.

In a second case, the patient was 32 years of age, and this was her first pregnancy—at the expiration of twenty-seven hours her delivery was completed—the child, a male, weighed 8¾ pounds. "It had apparently been dead for several hours."

In the third case, the mother, aged 24, was in her first pregnancy; after a labor of twenty-eight hours she was delivered of a male child, which weighed 7 pounds.

*Still-born.*—I find twenty-seven cases of delivery, in which the child was not born alive.

One of those was an instance of arrested development.

Five were cases of premature delivery.

In two cases, craniotomy was employed.

Three were delivered by the forceps.

One was a presentation of an arm and the funis.

Two were presentations of a foot.

One was a presentation of the breast.

In one the mother had constant convulsions for two hours previous to delivery.

One child was putrid when delivered.

In one case no physician was in attendance.

One was the second expelled of twins.

Of the remaining eight cases, two occurred in the first pregnancy, and six in the second pregnancy.

*Lacerated Perineum.*—I find but four cases of *lacerated perineum* spoken of, and these were all in the first labor.

In one case, the head was delayed a long time by the resistance of the external parts; the pains in the meanwhile were vigorous, and although the perineum was firmly supported, it suffered a slight laceration when the head finally passed. The records read, "it may be well to add that the patient was dwarfish and the child rather large." The child, a female, weighed 8½ pounds.

In a second case, where the forceps were applied, there was a slight degree of laceration, although the perineum was firmly supported.

In a third case, the laceration extended two-thirds of the space between the vagina and anus. It was probably torn by the passage of the shoulder, which immediately followed the head, while the perineum was unsupported in consequence of the accoucher's attention being directed to the condition of the umbilical cord which was protruded by the side of the head.

The fourth case of laceration occurred in a patient whose perineum was left unsupported to test the utility or inutility of such a course.

*Diseases.*—In the 451 cases of confinement, six cases of peritonitis occurred; three of convulsions; two of diarrhœa; two of utero-hemorrhage; one of phlegmasia dolens; one of ascites; one of neuralgia; one of typhus fever.

Neither of the cases of *convulsions* occurred during the period of my attendance, and, as I should injure the reports by an abstract, they are copied entire from the hospital records.* All the cases of *peritonitis* terminated favorably, as well as those of *hemorrhage;* that of *phlegmasia dolens;* that of *neuralgia;* and one of the cases of *diarrhœa.* While the case of *ascites,* one of the cases of *diarrhœa,* and the case of *ship fever,* were fatal.

*Child.*—With respect to the children, I find but little worthy of notice.

One case of *cephalœmatoma* occurred. The mother, aged 29 years, her fifth pregnancy. She was ten hours and a half in labor, and her child, a male, weighed eight pounds. The tumor was upon the right parietal bone. The mother left the hospital a fortnight after her confinement, and nothing is known of her child since.

In one case, death followed *haemorrhage from the cord.* The child, a female, weighing seven pounds and a half, was born after a labor of thirty-six hours. "During the afternoon, blood began to ooze from the extremity of the cord. It was tied again and again without effect."

On the next morning, "the right eye was livid and œdematous. Trunk and limbs œdematous, but not discolored ; also various parts, chiefly on limbs. The cellular tissue is almost of a stony hardness. The color of the skin not altered."

On the second day, "the cord has pulsated up to this time. Blood has continued to ooze from the *extremity* until to day ; it now flows from the root; the cord beginning to be detached. On the third day, the child died."

The only notices of *distortions* I can find are two instances in which "the right foot was turned inwards, varus."

To show that it is not an uncommon circumstance for a woman to be mistaken as to her pregnancy, I would state that, during the four years I was connected with this institution, three women of excellent character entered to be confined, neither of whom was pregnant. One of them, a very industrious Irishwoman, from the fact of her menses being suppressed, and her abdomen having become enlarged, and from the belief that she distinguished "quickening," made her usual calculations, expended all the means she could spare from her hard earnings to provide clothing for her expected offspring, and with her "permit," came to the hospital. I was called to attend her in her accouchment. She was in bed, expecting momentarily to be confined. Her pains were *false pains*—she was not pregnant.

Upon another occasion, I was called at night to attend two women who were expecting to be sick. One was in labor; the other, an American, had entered the house a few hours previously, and thought she should probably be confined during the night. While the labor of one was rapidly advancing, observing that the other was silent, I jocosely asked her what reasons she had for supposing she should be confined. She answered, "Because her doctor had told her she should be." I asked her if that were her only reason. She said it was a principal one; that her catamenia had been irregular, her abdomen had enlarged ; that she had suffered much pain in the region of the uterus ; and that her physician, satisfied of her pregnancy, had fixed upon the day of her delivery. I examined her, and found the uterus empty. She was suffering from a congestion of its neck, and was dismissed, to enter the Massachusetts General Hospital for treatment.

*They are omitted in this summary.—Ed. Buffalo Journal.

Instances of mistaken pregnancy are sometimes sufficently ludicrous. We were once summoned, in haste, to attend a case of labor, and on arriving at the house we met, before we entered the parturient chamber, two female neighbors who had been called to render assistance at the delivery. We found the patient primly sitting in an arm chair, presenting as spare and *collapsed* an appearance as could well be imagined within the limits of health. We were curious to inquire on what grounds she had fancied herself pregnant, and proceeded to ask if the menses had been interrupted, to which she replied that she had been perfectly regular. The only symptom of pregnancy, in fact, was, what she had imagined to be the motion of the child, but how she continued to consider this evidence sufficient, in spite of the most palpable absence of the least enlargement of the abdomen, was surprising ; and not less mysterious was the process by which she had arrived at the conviction that her period of delivery was at hand, not having the slightest approach to what could be mistaken for uterine pains.

The Report by Dr. Ramsay embraces an analysis of *four hundred and twenty-three cases* in private practice. Every case terminated favorably. The author distributes them in the following tabular arrangement:—

*Aggregate view, No.* 1.

| | | Frequency. | Per cent, |
|---|---|---|---|
| Natural Labor, - - - - - - | 5 cases twins, | 1 in 94,* | or 1.05 |
| Unnatural Labor, - - - - - - | 8 cases, | 1 in 59, | or 1.69 |
| Complex Labor, - - - - - - | 21 casss, | 1 in 22, | or 4.45 |
| Manual Labor, - - - - - - | 3 cases, | 1 in 157, | or 0.63 |
| Instrument. Labor, - - - - - - | 7 cases, | 1 in 67, | or 1.06 |

*Distinctive view, No.* 2.

EUTOCIA.

| Presentations. | No. | Numerical frequency. |
|---|---|---|
| Vertex,† - - - - - - - - - - | 429 | 429 in 473 |
| Feet,† - - - - - - - - - - | 3 | 1 in 157 |
| Breech, - - - - - - - - - - | 4 | 1 in 118 |
| Face, - - - - - - - - - - | 1 | 1 in 473 |

DYSTOCIA.

| | Presentations, etc, | No. of cases. | Frequency. |
|---|---|---|---|
| *Complicated, &c.* | Arm and shoulder, - - - - - | 3 | 1 in 157 |
| | Arm, foot and placenta, - - - | 1 | 1 in 473 |
| | Placenta previa, - - - - - | 2 | 1 in 236 |
| | Side and funis, - - - - - - | 1 | 1 in 473 |
| | Convulsions, - - - - - - | 4 | 1 in 118 |
| | Tumor, - - - - - - - | 1 | 1 in 473 |
| | Labial effusion, - - - - - - | 1 | 1 in 473 |
| | Hemorrhage, - - - - - - | 2 | 1 in 236 |
| | Adherent placenta, - - - - - | 1 | 1 in 473 |
| | Hour glass contraction, - - - | 3 | 1 in 157 |
| | Cord torn from placenta in utero, - | 1 | 1 in 473 |
| | Impacted head, - - - - - - | 1 | 1 in 473 |
| *Manual,*—Cases required version, | - - - - - - | 3 | 1 in 157 |
| *Instrumental,* | Forceps, - - - - - - - - - | 2 | 1 in 236 |
| | Required embryotomy or cephalotomy, - - | 5 | 1 in 94 |

* A general average with Clarke. Boivin and Baudelocque.
† Twin cases not included.

On comparison of the foregoing with the relative frequency of various forms of labor in French and English practice, as presented in statistics by Boudelocque, Boivin, Bland, Collins, and Merriman, the author deduces the following conclusions:—

1. That we do not have unnatural labors as frequently as the French and English, as far as our tables are capable of determining. 2d. That presentations have occurred more frequently to me, than to Baudelocque, Bland, or Collins. 3d. That puerperal convulsions have occurred oftener with me than with Collins or Merriman, although I have previously declared that our ladies enjoy almost an immunity, and attempted to account for it ; this unique position will find some atonement by recurring to Dr. Bland's experience in 1897 cases; and it will be fully explained in an annotation at another place. 4th. It will be seen that a lamentable deficiency exists in the tables of Boivin and Baudelocque, involving points of the highest practical and statistical magnitude. 5th. The mortality among French women will strike forcibly the most casual observer, when it is remembered that the forceps are seldom used, and the perforation sacrilegiously interdicted.

Dr. R. advocates the delivery of the placenta prior to the child, in cases of placenta prævia. With respect to the delivery of the placenta in other than placental presentations, he remarks:—"In my own practice I never have a retained placenta. I invariably deliver in 15 minutes after delivery, all things being fair. I have never had any cause to regret the practice, but the experience of every week proves to me its correctness. It is seldom I see a hemorrhage after delivery among my own cases, and I attribute it to the speedy delivery of the after-birth."

He expresses a belief in the hereditary influence of labor so far as *quickness or tediousness of the process is concerned.*

We have reproduced the foregoing additions to obstetrical statistics, in part from their intrinsic value and interest, and partly with a view to their suggesting the collection of data for similar enumerations by practitioners in different sections of the country. The comparison of statistical results contributed from various quarters, with each other, will be curious and useful; and these results aggregated, will furnish a representation of American obstetrical practice, which it will be interesting to compare with the statistics of foreign countries.

